# Efficient Ways to Combat Doping in a Sports Education Context!? A Systematic Review on Doping Prevention Measures Focusing on Young Age Groups

**DOI:** 10.3389/fspor.2021.673452

**Published:** 2021-12-16

**Authors:** Katharina Pöppel

**Affiliations:** Institute of Sport Science, Research Group “Sport and Education”, Carl von Ossietzky University of Oldenburg, Oldenburg, Germany

**Keywords:** athletic performance, doping prevention, anti-doping education, learning, literacy, systematic review

## Abstract

Youth is characterized by testing and crossing natural boundaries, sometimes with the help of performance-enhancing substances. In this context, doping prevention measures play a crucial role to protect individuals both within and outside the context of elite sport. Based on the PRISMA guidelines, a systematic literature search was conducted in the databases ProQuest (ERIC), Scopus, PSYNDEX/PsychInfo, PubMed, and Web of Science Core Collection to provide an overview of the impact of doping prevention measures, with particular attention to the underlying understanding of learning. As a result of the screening process, 30 of the initial 5,591 articles met the previously defined and recorded eligibility criteria. The analysis led to heterogeneous results regarding content, implementation, target group, or outcome variables considered relevant. Two-thirds of the studies related to the competitive sports context. Nevertheless, there has been a growing interest in studying doping prevention and its effects on non-elite athlete target groups in recent years. In terms of effectiveness, many measures did not achieve long-term changes or did not collect any follow-up data. This contrasts with understanding learning as sustained change and reduces the intended long-term protection of prevention measures, especially for adolescent target groups. Even young age groups from 10 years upwards benefited from doping prevention measures, and almost all doping prevention measures enabled their participants to increase their physical and health literacy. No conclusion can be drawn as to whether doping prevention measures based on constructivist ideas are superior to cognitivist approaches or a combination of both. Nevertheless, programs that actively engage their participants appear superior to lecture-based knowledge transfer. Most of the prevention measures offered a benefit-orientation so that participants can achieve added value, besides trying to initiate health-promoting change through rejection. Because of the lack of sustained changes, a further modification in doping prevention seems necessary. The review results support the value of primary prevention. Doping prevention measures should enable tailored learning and development options in the sense of more meaningful differentiation to individual needs. The implementation in a school context or an online setting is promising and sees doping as a problem for society. The review highlights the importance of accompanying evaluation measures to identify efficient prevention components that promote health and protect young people.

## Introduction

Doping prevention is a matter for society as a whole and not an exclusive concern of elite sport. This statement is the consequence of considering the desire for performance-enhancement as a societal phenomenon and acknowledging the association of athletic success and appearance with strength, competence, social ability, or beauty (Ahmadi and Svedsäter, 2016). Informed decision-making can therefore only take place if there is a sound basis, which is built up through targeted educational processes. When considering education in a sport and health context, it is necessary to address the needs and backgrounds of individuals to facilitate effective learning and achieve sustained outcomes. Young people, in particular, show an increased and possibly substance-assisted interest in optimization, both within and outside of competitive sports (Dunn and White, 2011). Since they naturally have a comparatively high level of physical performance, they seem more difficult to reach. However, since adolescents can also be affected by consequential health damage, prevention is indicated at an early stage. Based on a systematic literature review, this article summarizes the characteristics of successful prevention measures and derives implications for practice. This review is the first article that systematically examines doping prevention from a teaching-learning perspective to the author's knowledge. Thus, particular attention is paid to the content, the underlying understanding of learning, and the conditions of the learning environment.

### Education in Sport: From Promotion and Prevention

#### Educational Goals

Independent of the own starting point, the term literacy describes an overarching educational goal that everyone individually strives for, supported by appropriate learning opportunities. According to UNESCO's understanding, literacy means realizing one's potential and participation in society (Carr-Hill and Pessoa, 2008). Sports, including its rules and regulations, can be considered as a specific societal learning area. In the sports context, two literacy domains are of particular importance: Physical literacy (PL) and health literacy (HL). In summarized terms, PL encompasses physical, psychological, cognitive, and social learning domains that promote lifelong and holistic learning in movement contexts and an appreciation of movement for active, healthy living (Keegan et al., 2019). Accordingly, intending to optimize one's performance is a question of a reflective and conscious approach to one's physical possibilities and limits to remain active and healthy for as long as possible. Depending on the setting—e.g., physical education or elite sport—the impulses for acquisition are different in terms of quality and quantity. If we look at the goal of a healthy life mentioned there, a direct link to the construct of HL becomes apparent.

Bröder et al. (2019, p. 11) developed a working definition of HL for adolescents that “encompasses how health-related, multimodal information from various sources is accessed, understood, appraised, and communicated and used to inform decision-making in different situations in health (care) settings and contexts of everyday life while taking into account social, cognitive, and legal dependence.” HL is seen as the result of targeted health education and represents a vital social resource, including prevention and physical activity (Paakkari and Okan, 2020; Vamos et al., 2020). In summary, this means that both PL and HL have a cognitive and a physical component and that a successful learning process precedes literacy building. If one considers both constructs in the field of doping prevention, adolescents should be able to make independent, reasoned, prohibited, and health-promoting decisions to enhance their performance.

#### Effective Ways of Learning

Learning can be described as a permanent behavior change or ability to behave in a certain way that develops through exercise or experience (Schunk, 2012). As such, learning can be seen as an active, individually executed (developmental) process. Seen from a holistic perspective, it occurs in a network of society, parents, school, or peer groups (Darling-Hammond et al., 2020). When considering learning processes, young people represent a primary target group: (a) They have a favorable starting position for learning concerning cognitive (e.g., comparatively high degree of neural plasticity) and physical components (incl. health-related aspects), (b) They are integrated into institutional learning settings through their participation in school and physical education lessons, which may be supplemented by learning arrangements in organized sports, (c) They develop personal goals (possibly overestimating them), values and deal with finding an identity as a central developmental task for young people (Havighurst, 1948; Ghetti and Fandakova, 2020).

Referring to the WADA guideline for the International Standard for Education (ISE, World Anti-Doping Agency, 2021, p. 6), education is regarded as “The process of learning to instill values and develop behaviors that foster and protect the spirit of sport, and to prevent intentional and unintentional doping.” In terms of the understanding of literacy, doping prevention measures should support young people to use their acquired knowledge and understanding of their environment to make informed decisions (see Loland, 2017), e.g., how to be active in line with their performance limits and performance goals. In this context, educational researchers recommend a constructivist view of learning processes to support informed decision-making (Hanson, 2009). According to this, learning is conceived as the generation of meaning as a result of experience. Compared to behaviorism (learning as an observable change in behavior as a result of a stimulus, which also includes pure memorization) and cognitivism (learning as the acquisition of knowledge as a result of information processing and changes in cognitive structures), constructivist approaches appear superior as they evoke an active reflection of the object of learning (Ertmer and Newby, 2013). Problem-based learning can be seen as an example of a constructivist learning environment and represents a learner-centered approach through individual processing of real problems, like a confrontation with the use of performance-enhancing substances (Savery, 2006; Pöppel, 2020). The explanatory model of doping behavior determines which learning approach is considered adequate (Hauw and McNamee, 2015). Hanson (2009) takes a constructivist perspective and underlines two aspects for the creation and evaluation of measures in educational research in the field of performance optimization in sports: (1) Interventions should improve and potentially correct athlete's beliefs on performance-enhancing substances concerning their validity, and (2) Interventions should empower athletes with skills and critical attitudes to self-assess the outcomes of using performance-enhancing substances. According to the literacy concept and in the spectrum from school sports to elite sport, learning, and related performance goals differ (Table 1, see also Gilberg et al., 2006).

**Table 1:** Learning in sports.

**Setting/level**	**School/Physical education**	**Elite sport**
Physical performance goals	Defined by the curriculum for various sports, establishing a generally healthy habit	Optimization of physical performance in a specific sport, minimizing risk of injuries/health damages
Cognitive performance goals	Retrieval, application, and transfer of sport-related knowledge and literacy for the initiation/maintenance of an active and healthy lifestyle, including a critical appraisal of the associated aspects, including support options for physical performance	Development and application of sports related knowledge to create optimal performance conditions including creation and application of specific training plans, nutrition, support of recovery ability, etc.

It is evident that learning in sport goes beyond physical parameters and includes the promotion of personality development, moral integrity, or the (non-)use of performance-enhancing substances in the sense of education (Brand and Schwarz, 2017). These aspects are particularly relevant in adolescence and under increasing peer influence and pressure. It also implies the promotion of resilience development to deal with difficult situations (Smith-Osborne, 2007).

### Doping in Sports

When trying to reach the maximum of one's physical performance, a meaningful amount of people consider doping as a supportive aid. Petróczi (2021, p. S16) describes doping as a complex phenomenon in the differentiation between not-prohibited and prohibited attempts to foster performance: “Performance enhancement is encouraged in sport, provided it is achieved through legitimate means. When strategies for boosting performance employ substances or methods specifically outlawed by a governing body, such as the World Anti-Doping Agency (WADA), the practices become doping.” The aspect of prohibited rule-breaking behavior is crucial in competitive sports. However, athletes also try to improve their performance outside of competitive sports, such as recreational sports. In this context, doping (if one wants to use this term here as well to describe performance-enhancing activities) presents a public health concern and differs from a distinction in terms of prohibition. This is aggravated by the fact that the use of substances or technology to enhance performance outside of elite sport is also associated with positive values (Ahmadi and Svedsäter, 2016). Consequently, developers and implementers of doping prevention measures should consider this aspect (Petróczi et al., 2017). Accordingly, there is a difference in whether doping prevention occurs in a competitive or recreational sports context. It seems logical that prevention is also demanded outside of elite sport (e.g., Barkoukis et al., 2016).

#### Doping as a Performance Level Overarching Problem

Doping occurs inside and outside elite sports (Greydanus and Patel, 2010), including blurred boundaries in both settings. In amateur sport, we can observe doping in cycling (Henning and Dimeo, 2015) or triathlon (Dietz et al., 2013). As an aggravating factor, there is excessive and partly pain-preventive use of analgesics in soccer (Sachse and Steinberg, 2020) or triathlon, associated with an increased likelihood of using doping substances (Dietz et al., 2016; Seifarth et al., 2019).

In elite sport, the use of analgesics is also widespread and appears already among young athletes (Tscholl et al., 2008; Schneider et al., 2019). Additionally, one can describe doping as a severe elite sports problem. Some estimates range from 39.4 to 47.9% of international track and field athletes surveyed by means of randomized response technique who use doping substances (Ulrich et al., 2018). The willingness to take doping substances increases with age and is already evident among young elite athletes (Striegel et al., 2010). Based on a self-report, 75.3% of the athletes interviewed in a German questionnaire survey said they had thought about doping (Peters et al., 2009). Nevertheless, the data should be interpreted with caution. Gleaves et al. (2021) highlight a weak database and report prevalence rates ranging from 0 to 73% in the scope of a review. According to them, the reported prevalence shows geographic, sport-specific, population-specific (gender, age, level of competition), methodological, or definitory differences. Depending on the level of doping in one's sport, there may even be a normalization of doping in terms of the sport's perceived culture (Engelberg et al., 2015). It is therefore essential to examine what effects preventive measures of the anti-doping agencies achieve.

Both elite and amateur or recreational sports show increasing use of doping substances (Elbe and Barkoukis, 2017). Besides, analgesics aggravate the situation. These are in a gray area, and people may use them used with a performance-enhancing intention. Consequently, there is a need for effective doping prevention beyond the context of elite sport.

#### Adolescence as a Critical Doping Entry Phase

The studies on performance level-independent analgesics usage indicate a need to focus on young people. Doping already occurs in preadolescence, regardless of athletic performance level (Laure and Binsinger, 2005; Wanjek et al., 2007; Lucidi et al., 2008). Even under-10-years-olds report doping (Nicholls et al., 2017). One can assume that children cannot fully reflect on the consequences of their actions. Compared to the general population, more young people report using doping and having a higher affinity for other substances, such as alcohol or drugs (Dunn and White, 2011). In particular, young people appear to be susceptible to social norms in the form of performance expectations. Consequently, this may lead to an increase in the use of doping substances (Ahmadi and Svedsäter, 2016). Adolescence is characterized by specific attributes, like dedication to performance, including quick recovery after exhaustion, attractiveness, and sensation seeking or an optimistic bias about the risks of substance use (Arnett, 2000; Steinberg, 2007): This makes adolescents a particularly vulnerable group to at least try doping substances. Regarding negative (health) consequences and the extent, young people use substances to improve athletic performance in sports, it is apparent that there is a need to fight doping at a grassroots level (Barkoukis et al., 2016). Concentrating on the prevention of alcohol, drugs, or nicotine is therefore not enough. Thus, an early start to prevention is beneficial. In this case, there is the possibility of a primary preventive, positive orientation since doping behavior has not yet been shown or has not yet manifested itself over a more extended period (Singler, 2015).

#### Starting Points of Doping Prevention

Understanding doping and related risk factors is central to the design of anti-doping measures, as it determines the interventions' target components (Petróczi et al., 2017; Hurst et al., 2019). In a meta-analysis, Ntoumanis et al. (2014) identified a slightly adapted version of the Theory of Planned Behavior (TPB, Ajzen, 1991) as the best explanatory model to date for doping-related intentions and behaviors. In the course of their analyses, they found the comparatively strongest positive correlations between (a) use of not-prohibited supplements, b) perceived social norms, and (c) pro-doping attitudes and doping intentions and behaviors, whereas (d) morality and (e) self-efficacy to resist doping showed negative correlations with doping intentions and doping behaviors. Nevertheless, Petróczi et al. (2017) emphasize that the relationships between predictor and criterion should be regarded as weak, and consideration of further integrative and/or conceptual models is recommended to understand doping behavior better and to derive targeted prevention measures for specific target groups (Blank et al., 2016; Lazuras, 2016). Although the desire for clean athletes is at the center of efforts, less attention has been paid in research to this group, e.g., in terms of better understanding their clean sport identity or to further protect them from doping (Englar-Carlson et al., 2016; Petróczi et al., 2021).

#### Educational Settings as an Opportunity for Early Doping Prevention

Looking at doping prevention and its goals, it becomes clear that measures should be implemented before behavior onset as primary prevention and should be evaluated (Backhouse, 2015). Organized doping prevention by anti-doping agencies (e.g., Play True or ADEL of the WADA) focus mainly on the elite sport context and are mandatory for athletes. Focusing on young elite athletes and their experiences, most athletes had already taken part in doping prevention measures and described anti-doping education as a helpful tool. More positive effects were reported for measures offering more than pure information, highlighting the usefulness of multifaceted anti-doping efforts (Gatterer et al., 2021). Suppose one understands doping prevention as a societal concern (Petróczi et al., 2017). In that case, this means that many people vulnerable to performance-enhancing substance use are not reached by these prevention programs, especially adolescents outside an elite sport context. Meanwhile, doping prevention programs are offered more broadly and for performance heterogeneous target groups. One example is the SAFE YOU program funded by the European Union, which intends to address amateur and recreational sports athletes and offers a problem-based learning approach (www.safeyou.eu). Furthermore, anti-doping agencies offer targeted doping prevention measures for schools (e.g., sport values for every classroom, World Anti-Doping Agency, 2021). However, it is unclear whether these programs are used in the school context and what effects they achieve.

Educational settings in schools (e.g., physical education classes) offer a favorable opportunity because they appeal to young target groups regardless of their athletic performance. Unlike in elite sport, they do not need to involve personally addressed repressive information components such as punishments and suspensions. Taking Germany as an example, anti-doping education is not a compulsory part of the school's general physical education curriculum. It is only offered to students who wish to gain university entrance and have chosen physical education as an examination subject concerning their high school diploma (Ministry of Education Cultural Affairs of Lower Saxony, 2018). However, there is an effort to integrate anti-doping education into the German curricula in cooperation with the national anti-doping agency (Klüttermann, 2019). In other countries, too, prevention is not (yet) included in the physical education curriculum. Instead, the effects of doping on the body are considered, like in the Australian Curriculum for years 9 and 10. These contents are linked to critical and creative thinking, literacy, ethical understanding, lifelong physical activities, and games and sports (Australian Curriculum Assessment and Reporting Authority, n.d.). Consequently, the school context and the possibility of a cross-curricular perspective on doping prevention and the possibility of addressing vulnerable, adolescent target groups in an age-appropriate way offer opportunities to implement primary prevention measures with a broad focus on content.

### Learning Objectives in Doping Prevention

In 2021, WADA published the ISE as an overarching guideline for anti-doping organizations responsible for implementation in their respective countries. This publication can be labeled as “the most significant development in WADA's efforts” (Woolf, 2020, p. 8). When considering the learning objectives of doping prevention, one can use the distinction between a cognitive and an affective domain of the ISE (World Anti-Doping Agency, 2021), which, in addition to the focus on elite sport, also emphasizes a societal perspective (World Anti-Doping Agency, 2020). Table 2 breaks down the characteristics of ISE using Gatterer et al.'s (2019) content areas to analyze national anti-doping organizations' prevention efforts. The cognitive domain is characterized by knowledge development and thus includes an intellectual component. Looking at WADA's underlying hierarchy of learning qualities, namely remembering, understanding, and applying as well as analyzing, evaluating, and creating at the upper end of the hierarchy, a connection to the literacy concept becomes apparent (World Anti-Doping Agency, 2021). The guideline corresponds to a modern concept of doping prevention. It emphasizes the importance of active and multifaceted learning strategies, such as problem-solving/problem-based learning, critical thinking, or responsible decision-making, which can be aligned with a constructivist approach and usually require a face-to-face setting. Despite the proximity to elite sports, the importance of school-based learning settings is also underlined. The cognitive domain includes topics related to elite sport, such as the management of the World Anti-Doping Code (including the list of banned substances, sanctions for non-compliance), but also cross-cutting issues such as the long-term effects of doping substances on the body or nutrition (prevention as nutrition advice or health promotion). The latter topics are also relevant beyond the elite sport context. In this respect, the cognitive domain offers a cognitivist and a constructivist understanding of learning.

**Table 2:** Combined illustration of different learning understandings of doping prevention.

		**International Standard for Education (World Anti-Doping Agency**, **2021****)**			
		**Cognitive domain (remember, understand, apply, analyze, evaluate, create)**	**Affective domain (receiving, responding, valuing, organization, internalization)**
Categories described by Backhouse et al. (2014, p. 53) and Gatterer et al. (2019, p. 232)	Information	Knowledge-focused approach (e.g., prohibited list, side effects, consequences of doping)	
			Affective-focused approach (e.g., feelings of value and self-worth, self-image, personal challenges)
	Education	Social skills training (e.g., decision making under peer pressure, dilemma situations/resolve conflicts)	
		Life skills training (combination of social and personal skill and knowledge, e.g., decision making anticipating potential consequences)	
			Ethics and value-based (e.g., fair play, honesty, integrity)

For about a decade, scientists have discussed whether values-based approaches are more efficient (Backhouse et al., 2012). The affective domain includes the area of values-based education (e.g., moral education), as well as the areas of attitudes, motives, or emotions. Accordingly, World Anti-Doping Agency (2021) applies a broad understanding of the term and highlights the benefit of adding emotions for supporting learning. Aspects in the sense of personality development (prevention as the empowerment of self-esteem) can also be included here and can be directly related to adolescent's developmental tasks (Havighurst, 1948). Like the cognitive domain, the aim is to internalize the elements and actively implement them in daily life, both within and outside of elite sport, such as honesty, respect, or justice. In this respect, both program components show that WADA is pursuing a very broad-based prevention concept. A recent review examining 53 national anti-doping organizations' prevention efforts shows that knowledge-based approaches have dominated to date (Gatterer et al., 2019). However, the authors also recommend taking a broader perspective, including values-based approaches to anti-doping.

Two gender-specific programs are ATLAS (Adolescents Training and Learning to Avoid Steroids, Goldberg et al., 1996a) and ATHENA (Athletes Targeting Healthy Exercise and Nutrition Alternatives, Elliot et al., 2004), which were developed in the United States and targeted high school athletes. Both programs aim to promote healthy behaviors and reduce harmful behaviors (including doping, but also drug use or risky driving). The program curriculum consists of seven to eight sessions each, works with peer instructors, and actively involves the participants. Both programs are comprehensive examples of broad-based prevention measures in the school setting, including evaluation.

Recommendations for successful doping prevention go a content-based view: They include tailor-made measures adapted to the target group's needs and monitoring concerning implementation and achieved effects (Backhouse et al., 2012). Many prevention approaches aim to prevent behavior or change associated risk factors. Besides, some approaches recommend positive connotations and reinforce healthy behavior. Athletes who have been clean up to now should benefit from this in particular (Englar-Carlson et al., 2016). These attitudes are consistent with the understanding of primary prevention before the behavior was first exhibited (Singler, 2015).

Besides, evaluations are necessary to test doping prevention measures (Backhouse et al., 2012; Boardley et al., 2021). However, this is difficult. An evaluation of prevention measures in this sensitive field is complicated by restrictions in the openness of participants' responses and the need for reliable data. This difficulty is also evident in recording prevalence or a lack of reliable key indicators (de Hon et al., 2015). In order to achieve more robust results, scientists suggested indirect methods, e.g., projective questioning (questioning about other people to draw conclusions about the person), network scale-up (querying the proportion of trait carriers in relation to a specific whole), hypothetical situations (e.g., dilemma situations) or the consideration of reaction times in specific tasks (Petróczi, 2016). However, one may question whether an evaluation of the impact of prevention interventions is too late in the process, and instead, one should take a more informed look at the design of prevention interventions. For example, Woolf (2020) questions whether new prevention efforts, such as ISE, can lead to particular advances. In particular, he criticizes a lack of consideration of research in education and related disciplines since knowledge in itself does not lead to a change in behavior.

### Review Aims

While some studies have focused primarily on providing an overview (see a research analysis by Sipavičiute et al., 2020) or looking at the effect of doping prevention interventions (see a review by Bates et al., 2019), less attention has been paid to the underlying understanding of learning. This is remarkable because successful learning processes can be seen as an essential prerequisite for progress in anti-doping efforts. Therefore, this article intends to systematically review studies dealing with the evaluation of doping prevention measures to draw conclusions on beneficial learning strategies and implementation options in light of the effects achieved. With particular attention to adolescence, the underlying research question is: How should effective doping prevention measures be designed for young target groups, and (if data permits) what effect do doping prevention measures have on their participants? This question refers to three sub-areas, which are considered more specifically to identify features of successful doping prevention measures:

1. Characteristics of the target group
2. Characteristics of prevention measures in terms of their content
3. Characteristics of their implementation (e.g., learning comprehension or time scope).

In addition to previous recommendations, further practical implications are derived from the results of this review.

## Methods

The systematic review was conducted following the PRISMA recommendations (Moher et al., 2009). Pre-registration with OSF was carried out before the start of implementation (Pöppel, 2021). In addition to the pre-uploaded examination framework, further decision-making steps can be viewed there to increase transparency.

### Search Strategy

A comprehensive search was carried out in December 2020 in the databases ProQuest (ERIC), Scopus, PSYNDEX/PsychInfo, PubMed, and Web of Science Core Collection databases. The search strategy was based on a specification of the thematic content of relevant studies, the population studied, the label of the intervention, including a description of possible outcomes, and references relating to the studies' evaluation. The systematic approach and the search terms used are presented in Table 3. If possible, the search was limited to the consideration of the search terms in the title, abstract, or keywords and English or German language publications. No time limit was specified so that studies published at any time and up to December 2020 could be considered. Two additional steps supplemented the search: (1) a search within the framework of the German sports-specific database SPONET as well as GoogleScholar, and (2) the use of the snowballing technique (Greenhalgh and Peacock, 2005) related to a review of the literature lists of papers after application of the eligibility criteria. The results management was organized *via* the reference management software EndNote.

**Table 3:** Search strategy used.

**Doping**	**Population**	**Intervention**	**Outcome**	**Evaluation**
Doping	Athlet*	Intervent*	Prevent*	Evaluat*
Performance-enhanc*	Sport*	Educat*	Reduc*	Success*
Performance enhanc*	School*	Program*	Health	Effective*
Illicit	Adolescent*	Treatment*	Improv*	Measure
Prohibited	Coach	Campaign	Decreas*	Examin*
	Entourage	Anti-doping	Increase*	Assess*
	Elite	Anti-doping	Change*	Compar*
	Youth	Anti-doping	Stop*	
	Pupil*	Measure	Refus*	
			Avoid*	
			Protect*	
			Combat	
			Fight	

* *Truncation character*.

### Inclusion Criteria and Study Selection

**Figure 1:**
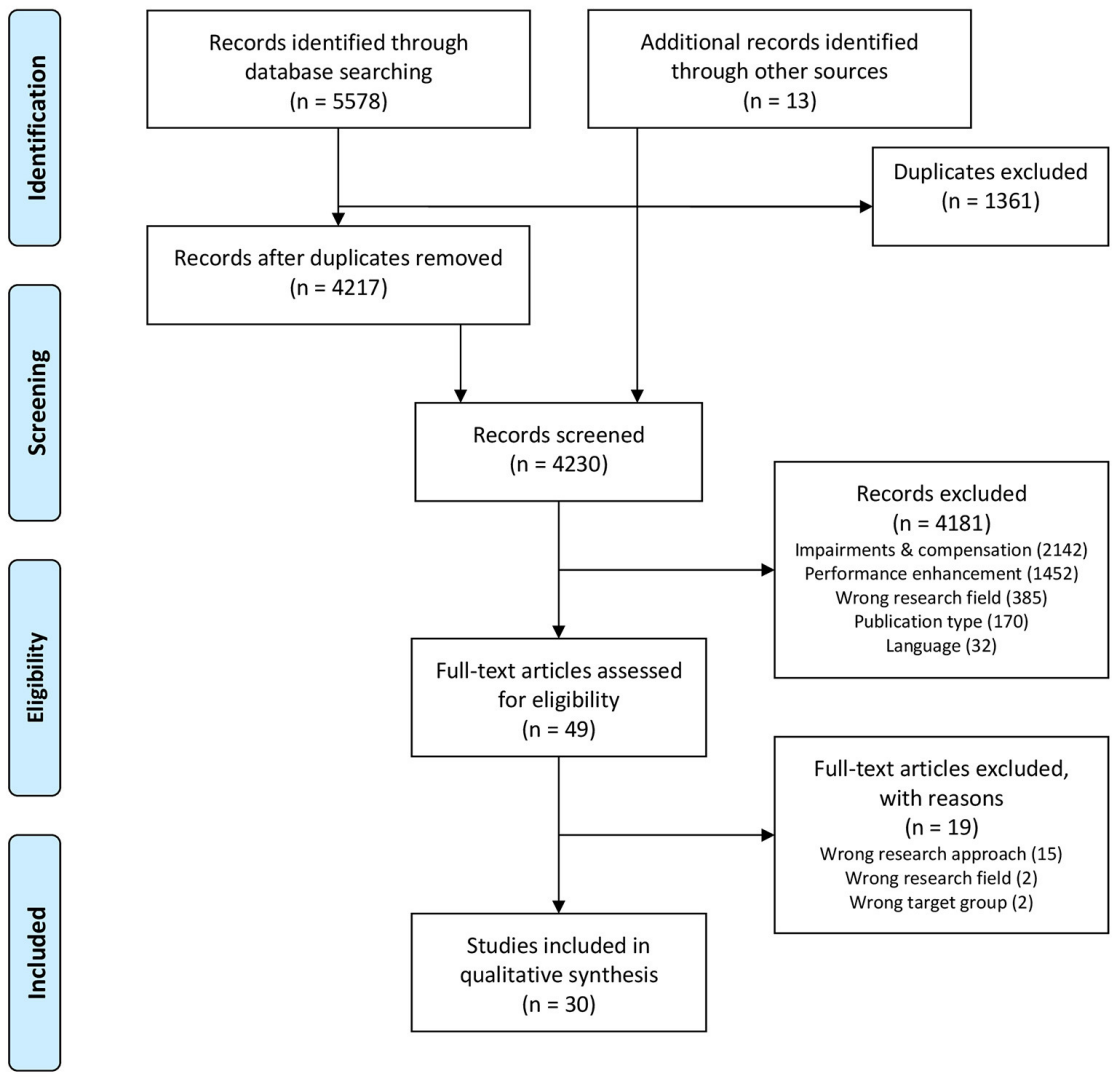
PRISMA flow diagram for identifying and selection studies evaluating doping prevention.

The definition of the eligibility criteria was based on the PICOS approach to specifying the participants, interventions, comparisons, outcomes, and study designs to be considered in advance (Moher et al., 2009). Inclusion criteria related to the participants were the consideration of all age groups (particular focus on adolescent age and young adults) as well as persons inside (elite) sports such as athletes and persons outside (elite) sports such as students. Studies were excluded with people who had been asked about prevention measures but had not participated themselves. Concerning the intervention, studies were considered that relate to self-contained doping prevention measures or measures aimed at reducing the use of body/performance-enhancing substances or measures that aimed to develop a reflective attitude in this respect. Furthermore, interventions could include a broader spectrum of topics such as literacy (e.g., health or media), self-esteem, or moral/values. There was no restriction regarding the intensity or supervision of the measure. Accordingly, studies were not considered eligible if they assessed doping-related variables (e.g., motives for use, attitudes, intention to use) independently of a specific prevention measure or studies dealing with the willingness to participate in a doping prevention measure. In terms of comparisons, studies were considered eligible when contrasting participants with and without a prevention measure or comparing different variants of a measure. Eligibility includes control group designs as well as experimental group-only comparisons as well as qualitative considerations. Studies were defined as not eligible if they encompassed doping prevention-related study data of persons obtained outside the investigation of specific doping prevention measures. The outcomes considered are already defined in the context of the review aims. Concerning study design, studies were considered eligible if they described the effect of doping prevention measures, if they were quantitative, qualitative, or mixed-methods designs and if the study was published in a peer-reviewed English or German language journal. Accordingly, non-empirical statements, non-peer-reviewed publications including books, book sections, Ph.D. thesis, and single case studies were excluded from the analysis. The two-phase screening process started with an examination of the title and abstract. If these parts were considered eligible, an analysis of the entire text followed (see Figure 1, which visualizes the entire screening process).

### Data Extraction or Quality Assessment

Risk of bias assessment and thus evaluation of the methodological quality of studies was based on the CONSORT checklist (Schulz et al., 2010) and the Mixed Methods Appraisal Tool (Pluye et al., 2011). Each study's strengths and weaknesses were assessed using eleven criteria, and the results were displayed using a rating system ranging from weak (1) to strong (4). The quality assessment is displayed in Table 4.

**Table 4:** Evaluation of the methodological quality of the studies considered to assess the risk of bias (based on Schulz et al., 2010; Pluye et al., 2011).

**Author(s)**	**1**	**2**	**3**	**4**	**5**	**6**	**7**	**8**	**9**	**10**	**11**	**Sum**	**%**	**Score**
Barkoukis et al. (2016)	Y	Y	Y	Y	N	Y	Y	Y	P	N	N	7.5	68	***
Codella et al. (2019)	Y	Y	P	N	N	N	N	N	Y	N	Y	4.5	41	**
Duncan and Hallward (2019)	Y	Y	Y	P	N	Y	Y	Y	P	Y	Y	9	82	****
Elbe and Brand (2016)	Y	Y	Y	P	N	Y	Y	Y	P	P	Y	8.5	77	****
Elliot et al. (2008)	P	Y	Y	P	N	N	Y	Y	P	N	N	5.5	50	**
Elliot et al. (2006) → (see Elliot et al., 2004)														
Elliot et al. (2004)	N	Y	Y	P	N	Y	Y	Y	N	N	N	5.5	50	**
Goldberg et al. (2000) → (see Goldberg et al., 1996a)														
Goldberg et al. (1996a)	Y	P	Y	P	N	Y	P	Y	P	P	N	6.5	59	***
Goldberg et al. (1996b)	Y	Y	Y	P	N	N	P	P	P	N	N	5	45	**
Goldberg et al. (1991)	N	Y	P	N	N	N	P	Y	P	N	N	3.5	32	**
Goldberg et al. (1990)	N	Y	Y	P	N	N	Y	Y	P	N	N	5	45	**
Halliburton and Fritz (2018)	P	Y	N	P	N	N	P	Y	Y	Y	Y	6.5	59	***
Horcajo et al. (2019)	Y	Y	Y	Y	Y	N	Y	Y	Y	Y	Y	10	90	****
Horcajo and de la Vega (2014)	Y	Y	Y	Y	N	Y	P	Y	Y	P	N	8	73	***
Hurst et al. (2020)	Y	P	Y	Y	N	P	Y	N	P	N	Y	6	54	***
Jalilian et al. (2011)	Y	P	P	Y	N	N	P	Y	N	N	Y	5.5	50	**
Laure et al. (2009)	Y	Y	Y	P	N	N	Y	Y	Y	P	N	7	63	***
Lucidi et al. (2017)	Y	Y	Y	N	N	Y	Y	N	Y	P	Y	7	63	***
MacKinnon et al. (2001)	Y	Y	P	Y	N	Y	P	Y	Y	P	N	7.5	68	***
Mallia et al. (2020)	Y	Y	Y	Y	N	P	N	N	Y	P	Y	7	63	***
Medina et al. (2019)	P	Y	Y	N	N	N	P	N	P	N	N	3	27	**
Nicholls et al. (2020)	Y	Y	Y	Y	N	P	Y	Y	Y	N	Y	8.5	77	****
Nilsson et al. (2004)	Y	Y	P	P	N	N	Y	N	N	N	N	4	36	**
Ntoumanis et al. (2020)	Y	Y	Y	Y	Y	N	Y	Y	Y	N	Y	9	81	****
Ranby et al. (2009)	Y	Y	Y	P	N	Y	Y	Y	Y	P	Y	8	73	***
Sagoe et al. (2016)	Y	Y	P	Y	N	N	Y	Y	P	N	N	6	54	***
Wicki et al. (2018)	Y	Y	N	P	N	N	P	N	Y	N	N	4	36	**
Wippert and Fließer (2016)	P	Y	N	N	P	N	P	N	N	N	Y	3.5	32	**
Yager et al. (2019)	Y	Y	Y	Y	N	N	P	Y	P	Y	N	6.5	59	***

*1: [Introduction] Scientific background and explanation of rationale. Y, precise scientific background; P, brief overview; N, not specified*.

*2: [Introduction] Specification of a research question, specific objectives and/or hypotheses. Y, yes; P, implied; N, not specified*.

*3: [Methods] Information concerning the intervention (especially regarding: content, implementation, transparency/sufficient details for replication. Y, yes; P, implied; N, not specified*.

*4: [Methods] Completely defined pre-specified primary and secondary outcome measures, including how and when they were assessed; fit of collected data and research question. Y, measures specified beforehand, validated and reliable (Cronbach's alpha > 0.80); P, measure specified beforehand, but weaknesses concerning measures applied (e.g., single-item, Cronbach's alpha <0.80); N, measures not specified appropriately, regardless of the quality of the measure used*.

*5: [Methods] Determination of the sample size before conducting the study. Y, yes; N, not specified*.

*6: [Methods] Minimization of a selection bias in the recruitment of participants. Y, indication of why the selected sample is considered representative; P, sample representative with limitations; N, not reported or disregarded*.

*7: [Methods] Representativeness of the participants with regard to study goal. Y, yes; P, partly; N, no;*.

*8: [Methods] Information about sample composition, e.g., randomization. Y, yes; N, no*.

*9: [Results] Statistical methods used to compare groups for primary and secondary outcomes, completeness of information (e.g., checking for distribution violation before using metric procedures). Y, yes; P, partly; N, no*.

*10 [Results/Discussion] Significance of results/limits, e.g., low drop-out below 20%. Y, yes; P, partly; N, no or lack of report*.

*11 [Acknowledgments] Conflict of interest. Y, no conflict of interest/criterion met, N, conflict of interest (incl. reviewed conflict of interest) or not specified/criterion not met*.

*Score*:

* *(lowest quality)*,

**, ***, **** *(highest quality)*.

### Data Analysis

Due to the heterogeneity of the individual studies concerning design, analysis, and limited statistical parameters, the analysis is carried out on a narrative level. The systematics of the presentation is based on an extraction of the understanding of learning, including implementation and a comparison of the outcomes to be able to conclude the conditions for success.

## Results

After the removal of duplicates, 4,230 studies were identified through the database and supplementary search. As shown in the flow diagram (see Figure 1), the selection process resulted in 30 studies used for consideration in this review.

### Description of Studies Considered

The studies considered cover the years 1990–2020 and indicate a growing interest in doping prevention and young target groups. This trend is particularly evident in the last five years (53% of the studies were published between 2016 and 2020). The studies were mainly conducted in western societies (53% Europe, 37% USA) and span a performance spectrum from international elite athletes (Hurst et al., 2020) to students with no specific sport backgrounds (e.g., Mallia et al., 2020). The main target groups are athletes from different performance levels (63% of the studies). Nevertheless, there is a trend toward addressing more non-athletic target groups, such as students (33%). Mostly recreational athletes or young people integrated into a sporting context served as a target group in these cases. Although this setting differs from an elite sport context, the authors highlight the necessity of a low-threshold service focusing on health promotion and a general anti-doping mindset (e.g., Laure et al., 2009). The studies were heterogeneous regarding their content, implementation, target groups, and outcome variables.

All of the articles demonstrated a consideration of the state of research. The presentation of the respective study background was evidence-based, albeit sometimes superficially and with global reference to other prevention measures (e.g., Wippert and Fließer, 2016; Wicki et al., 2018). In half of the prevention measures described, no theory is apparent that was used to derive the procedure. In 22.7% of the prevention measures, the TPB (Ajzen, 1991) was mentioned, partly referencing that it is frequently used to explain doping behavior, but without any specific derivation of the prevention measure. A link to the TPB (Ajzen, 1991) could be identified in numerous studies concerning the outcome variables considered, namely doping behavior, intention, attitudes, norms, and perceived behavioral control.

In terms of content, almost all prevention measures conveyed knowledge about doping or related directly to the topic of doping. Only a small number of studies used alternative labels, such as media literacy (Lucidi et al., 2017; Mallia et al., 2020) or supportive communication strategies for coaches (Ntoumanis et al., 2020) to address anti-doping in a related and more indirect way. Nevertheless, learning and development opportunities for increasing one's PL and HL could be identified in almost all studies. Only the Clean Sport Program (Hurst et al., 2020) focused on competitive sport-related topics without health-related benefits, such as ten anti-doping rule violations, the process of doping control, testing of medicine for a not-prohibited use, or the danger of contaminated supplements.

Most doping prevention measures took place in a face-to-face setting. Only two studies conducted online-based prevention measures (Elbe and Brand, 2016; Nicholls et al., 2020). Based on the underlying quality rating of the studies from weak (1) to strong (4), the analyzed studies are on a medium level with *M* = 2.79 (*SD* = 0.78). One of the notable features here is that only a few studies have previously conducted a power analysis to determine the optimal sample size (Horcajo et al., 2019; Ntoumanis et al., 2020). Table 5 provides a study-oriented overview of the study results.

**Table 5:** Summary of included studies.

			**Literacy**		**Learning** **comprehension**			**Implementation**			**Outcome**																	*
**References [Country]**	**Progam [design]**	**Sample**	**PL**	**HL**	**cog**	**con**	**val**	**Scope**	**Method**	**Targeting doping**	**a**	**b**	**c**	**d**	**e**	**f**	**g**	**h**	**i**	**j**	**k**	**l**	**m**	**n**	**o**	**p**	**q**	
Barkoukis et al. (2016) [Greece]	Anti-doping culture promotion (health, values, nutrition, doping) [RCT/pre-post]	218 non-athlete adolescents	X	X	X	X		10 × 90 min/weekly	I, G	Direct			–						S				S				–	3
Codella et al. (2019) [Italy]	*Lotta al doping*: Anti-doping culture promotion (doping knowledge including moral and ethical aspects) [pre-post]	20,800 non-athlete adolescents	X	X	X			2-h seminar	I, G	Direct					S							S						2
Elliot et al. (2004, 2006, 2008) and Ranby et al. (2009) [USA]	*ATHENA:* Health promotion (substance use, healthy sport nutrition, effective exercise training, connection of behavior and performance, media perception, depression) [RCT/pre-post-9 month follow up]	928 resp. 2,092 female adolescent athletes	X	X	X	X		8 × 45 min/weekly	I, P, G	Direct	S	L			S			S	S L		S	S			S	S		2
Goldberg et al. (1990) [USA]	Educational intervention (information on adverse effects and limited effects of substance use) [RCT/pre-post]	190 male adolescent athletes	X	X	X			20 min talk + handout	I	Direct			–		–	S												2
Goldberg et al. (1991) [USA]	Fear-based intervention (information on adverse effects of AS) [RCT/pre-post]	191 male adolescent athletes	X	X	X			20 min talk + handout	I	Direct			–		–	–												2
Goldberg et al. (1996b) [USA]	*ATLAS* (pilot): Health promotion (steroid and nutrition education, strength training) [CBA/pre-post]	90 male adolescent athletes	X	X	X	X		8 × 60 min/weekly + 8 × weight room	I, P, G	Direct		–	–	S	S	S	–			S			–					2
Goldberg et al. (1996a), Goldberg et al. (2000), Halliburton and Fritz (2018), and MacKinnon et al. (2001) [USA]	*ATLAS* (Cohort 1–3) 1996 and 2018: cohort 1 2000: cohort 1–3, follow up: cohort 1,2 [RCT/pre-post-9 or 12 month follow up]	1,506 resp. 3,207 male adolescent athletes	X	X	X	X		Cohort 1: 7 × 50 min/weekly +7 weight room Cohort 2,3 8 sessions (5 × class + 3 × weight)	I, P, G	Direct	S L	S L			S L	S L	S L	L		S L	S L				S L			3
Yager et al. (2019) [Australia]	*ATLAS* (Application) [CBA/pre-post]	221 non-athlete adolescents	X	X	X	X		5 × 90 min/twice weekly	I, P, G	Direct	–	–	–		S			–	–	S								3
Elbe and Brand (2016) [Germany]	Ethical decision-making training (decision making in dilemma situations) [CBA/pre-post]	69 adolescent elite athletes	X	X			X	6 × 30 min		Direct, online			N															4
Hurst et al. (2020) [UK]	*Clean Sport* Educational intervention (doping and supplement knowledge) [pre-post-3 month follow up]	332 young elite athletes	X		X			60 min session	I	Direct	S		S		S L				S L	S L							S	3
Jalilian et al. (2011) [Iran]	Doping education (refusal skills, nutrition, training) [CBA/pre-post]	120 young male gym users	X	X	X	X		6 × 60 min	I, G	Direct		S	S		S			S					–	S				2
Laure et al. (2009) [France]	Life skills-based anti-doping intervention (self-assertion, doping knowledge, medication) [CBA/pre-post]	760 adolescents	X	X	X	X		2 × 2 h	I, G	Direct							S											3
Lucidi et al. (2017) [Italy]	Health promotion *via* media literacy intervention (performance and/or esthetic goals) [CBA/pre-post]	389 adolescent non-athletes	X	X	X	X		12 × 90 min	I, G	Indirect	–	–	S					S					–					3
Mallia et al. (2020) [Italy]	Health promotion *via* media literacy intervention (performance and/or esthetic goals) [CBA/pre-post]	521 sport science students	X	X	X	X		12 × 90 min	I, G	Indirect			–															3
Medina et al. (2019) [Spain]	Anti-Doping Education (knowledge, beliefs and attitudes, including values) [CBA/pre-post]	540 adolescent non-athletes	X	X	X	X	X	6 × 55 min/twice weekly	I, G	Direct			S	S	S	–												2
Nicholls et al. (2020) [UK]	*iPlayClean* (Health and personality promotion: doping and nutrition knowledge, developing sportsperson ship, resistance) [RCT/pre-post-4 month follow up]	1,081 young elite athletes	X	X	X			10 × 90 min/weekly	I	Direct offline and/or online			S F				S											4
Nilsson et al. (2004) [Sweden]	Appearance and social norms focusing program for male adolescents [cross-sectional study]	541 male adolescent non-athletes	X	X	X	X		12 × lectures + small group training + info material	I, G	Direct	–		–															2
Ntoumanis et al. (2020) [Australia, UK, Greece]	*CoachMADE:* Communication-focused anti-doping education for coaches (creation of supportive motivational atmosphere, need supportive communication) [RCT/pre-post-2 month follow up]	919 young athletes	X	X				2 work-shops for coaches	I	Indirect		S	–		L		–										–	4
Sagoe et al. (2016) [Norway]	*Hercules*: Health promotion (anti-doping education, strength training) [RCT/pre-post]	202 high school students	X	X	X			4 × 90 min session theory (with 12 workout sessions)	I	Direct		–	–		S	S	–				S				S			3
Wicki et al. (2018) [Switzerland]	*Cool and Clean* Emotional access to topics instead of pure knowledge transfer to enhance personal responsibility inside and outside sports (e.g., doping) [cross-sectional study]	1,887 adolescent athletes	X	X		X		Not specified	I, G	Direct and Indirect			S															2
Wippert and Fließer (2016) [Germany]	NADA Anti-doping program (doping education and personal development) [cross-sectional study]	213 adolescent elite athletes	X	X	X	X		Talk and/or full day seminar	I	Direct					L		–											2
Duncan and Hallward (2019) [Canada]	Impact of gain- vs. loss-framed messages [RCT/pre-post]	133 young athletes						Impact of message		Direct		–	–				–						–					4
Horcajo and De La Vega (2014) [Spain]	Impact of (anti-) doping message (risk vs. benefits of doping legalization) [RT/post-1 week follow up]	68 young athletes						Impact of message		Direct			S															3
Horcajo et al. (2019) [Spain]	None Impact of (anti-) doping message (risk vs. benefits of doping legalization) [RT/pre-post]	136 university students						Impact of message		Direct			S															4

*PL, physical literacy; HL, health literacy; cog, cognitivism; con, constructivism; val, values-based; a, doping behavior; b, doping intention; c, doping attitude; d, doping belief; e, doping knowledge; f, doping health risk/side effect knowledge; g, resistance against offer; h, supplement use; i, supplement attitude/intention; j, supplement knowledge; k, nutritional behavior; l, nutrition knowledge; m, norms; n, perceived behavior control; o, self-efficacy strength training; p, not-prohibited performance enhancing pill use; q, values of sport/moral disengagement*;

* Quality rating of the studies (*1/weak; **2; ***3; ****4/strong), X, applicable; P, Peer-led method; I, instructor-led method; G, group work of participants; S, positive short term effect (pre-post); L, positive long-term effect; N, negative effect; –, no meaningful difference; NADA, National Anti-Doping Agency Germany.

### General Effects of Doping Prevention Measures

Nineteen studies provided only pre-post or cross-sectional data. Therefore, no statement about long-term effects is possible, and one cannot conclude whether a sustained learning effect could be achieved. As an outcome, the studies mainly considered directly doping-related variables (e.g., doping behavior, intention, attitude, belief, knowledge) or associated variables, such as nutritional supplements (e.g., use, knowledge, intention) or diet. Less frequently, norms, behavioral control, or values were evaluated. Concerning self-reported doping behavior, it is noticeable that studies that surveyed both behavior and doping intentions and attitudes showed short-term but rarely long-term changes in doping intentions or attitudes (Goldberg et al., 1990; Ranby et al., 2009; Lucidi et al., 2017; Hurst et al., 2020). However, these changes were not or only in the short term associated with a behavior change (Goldberg et al., 1996a; Lucidi et al., 2017; Hurst et al., 2020). An exception was the three-cohort summary evaluation of the ATLAS program (3,207 athletes), which indicated short- and long-term improvements in both doping intention and doping behavior (Goldberg et al., 2000). If the prevention programs ATLAS and ATHENA are combined in the evaluation, small but significant changes could be demonstrated (Ntoumanis et al., 2014). This result contrasts with an application of the ATLAS program that does not show changes at either the intention, attitude, or behavioral level (Yager et al., 2019). Based on initial critical doping intention or attitude scores, some studies pointed to a floor effect to account for lack of change (Ntoumanis et al., 2014; Lucidi et al., 2017; Yager et al., 2019).

Three studies stand out in this evaluation because they do not examine a doping prevention measure but look at the effect of doping-related messages (Duncan and Hallward, 2019; Horcajo et al., 2019, 2020). These interventions were not analyzed in consideration of doping prevention interventions. However, they met the inclusion criteria and showed that even brief exposure to the issue of (not) legalizing doping could evoke change. These studies were presented separately (see the lower part of Table 3). For more details related to doping prevention measures' effectiveness, see the review by Bates et al. (2019), which considers studies up to 2016.

### Results in the Context of the Target Group

It is noticeable that programs for adolescent non-athletes were often placed in the educational context, like school. Here, adolescents around the age of 15 were the primary target group, and less frequently, programs targeted younger audiences, such as 10-year-olds (Laure et al., 2009) or 12–13-year-olds (Medina et al., 2019). Independent of the age group, this allows for the inclusion of a heterogeneous target group with potentially equally heterogeneous interests. The studies by Laure et al. (2009) and Medina et al. (2019) show that positive short-term effects could also be achieved in younger age groups. However, both studies do not allow any statement about long-term effects due to their pre-post design.

Especially in recent years, there has been a trend toward considering non-athletic target groups (e.g., Barkoukis et al., 2016; Medina et al., 2019). Knowledge about banned substances necessary for the elite sport was replaced or supplemented outside of competitive sports by more general doping knowledge, especially with a health focus. In the case of young non-athletes, the programs also considered nutrition, values, or aspects of personality development, which appear to be central irrespective of performance level. Accordingly, the overarching goal can be seen as enabling a healthy and active life.

Comparing the intended effects of prevention programs and the lack of effects, broken down by athletic and non-athletic target groups, shows that athletes were more likely to benefit from prevention. Simultaneously, the picture was relatively balanced between effects and lack of effects among non-athletic target groups. Nevertheless, non-athletes also benefit from prevention, although not to the same extent as athletes.

### Impact of the Content of Doping Prevention Measures

The approach to doping prevention varied considerably between the studies. A large proportion of the studies impart knowledge about doping or related topics (e.g., side effects or long-term consequences). Besides, a large part of the prevention measures also offered a benefit character. For example, the ATLAS and ATHENA programs imparted knowledge about healthy eating or efficient training (Goldberg et al., 2000; Elliot et al., 2008), or iPlayClean intended to promote resilience (Nicholls et al., 2020). Thus, most prevention measures offered a relatively balanced relationship between privation (e.g., reducing substance use to optimizes) and bonus (e.g., ways to improve performance *via* nutrition). Programs that provided a personal benefit through the topics covered appeared to be more effective. Meanwhile, measures that focused purely on deterrence (Goldberg et al., 1990) or focused on adverse effects (Goldberg et al., 1991) have disappeared in scientific evaluations. These approaches were also not convincing in terms of their effectiveness. The data suggest that imparting knowledge alone is not decisive for the success of a program.

### Impact of the Implementation of Doping Prevention Measures

Continuing the aforementioned part of the analysis more in-depth, the programs are classified according to the systematics of Gatterer et al. (2019) (see Table 2), with an additional assessment of the apparent learning understanding. Both components are presented separately since the classification is primarily content-oriented. It can be deduced that the knowledge-focused approach is associated with a cognitivist understanding of learning and thus shows a close connection to the cognitive domain of the ISE (World Anti-Doping Agency, 2021), while the areas of social skills training and life skills training can be assigned to a cognitivist approach. Since there is no corresponding affect-oriented learning theory, the affective-focused approach and ethics and value-based approach are understood as affective domain according to the ISE denomination of the World Anti-Doping Agency (2021).

Most prevention programs do not fall into a single category. Three exceptions can be identified, leaving aside the outdated purely knowledge-based information sessions on side effects (Goldberg et al., 1990) and fear-based intervention (Goldberg et al., 1991): (1) the ethical decision-making training (Elbe and Brand, 2016), which has a clear assignment to the affective domain and can be classified as ethics and value-based, (2) the CoachMADE program (Ntoumanis et al., 2020), which can be assigned to the social skills training category by promoting the communicative skills of coaches and aims to improve interaction with their athletes; and (3) the Cool and Clean program (Wicki et al., 2018), which–like the category–is designated as life skills training. Therefore, the last-mentioned program can be assigned to a constructivist understanding of learning, and it represented the only program that focused primarily on this approach. The program's core elements are promoting interpersonal and intrapersonal skills to support adolescents to cope with challenging situations.

Even the programs that can be assigned mainly to the cognitive domain and knowledge-based focus offer an additional component, e.g., combined with strength training (ATLAS by Goldberg et al., 1996b; Hercules by Sagoe et al., 2016), the inclusion of moral and ethical aspects (Lotta al doping by Codella et al., 2019), or development of resistance (iPlayClean by Nicholls et al., 2020). In these cases, the information provided within the article was superficial, that no assignment to a category beyond knowledge-based focus was carried out. A large proportion of prevention programs combine the knowledge-based component with social skills training (e.g., Jalilian et al., 2011; Hurst et al., 2020) or life skills training (e.g., Laure et al., 2009; Wippert and Fließer, 2016; Lucidi et al., 2017). Only a few prevention programs consider aspects of the affective domain. Elements of the affective-focused approach can only be found in Nilsson et al. (2004), whose approach explicitly attempts to increase self-awareness in addition to knowledge-focused components and elements of social skills training. In the case of two programs, which both aim at target groups outside the field of competitive sports, one gets the impression that they contain a little bit of everything. Medina et al.'s (2019) description considers elements of the knowledge-focused approach, life skills training, and ethics and values. In this case, general values of sports–like fair play–were addressed. The approach described by Barkoukis et al. (2016) additionally contains elements of social skills training. Based on this systematic, no category or combination of categories is particularly successful in measuring program success.

Regardless of the content areas covered by the programs, the final step is a detached examination of learning comprehension. In about half of all prevention measures, both cognitivist and constructivist components can be identified. Programs were found to be constructivist if they included, for example, problem-solving, decision making, investigative activities, creation of own anti-doping campaigns, or group debates (Barkoukis et al., 2016; Lucidi et al., 2017; Medina et al., 2019). As can already be seen in consideration of content, one cannot draw a clear conclusion whether cognitivist doping prevention approaches or a combination of constructivist and cognitivist approaches offered a more meaningful benefit for participants. Even when comparing activating programs (incl. group work) and passive programs (e.g., lectures), no clear difference can be seen. In comparing positive program effects and programs leading to no effects (concerning the variables examined), there was only a small difference in favor of programs characterized by active components.

The temporal scope of individual prevention interventions shows an enormous range between 20 min (Goldberg et al., 1990, 1991) and 18 h (Lucidi et al., 2017; Mallia et al., 2020). More comprehensive interventions in terms of the time were not associated with more positive effects. The 60-min Clean Sport program (Hurst et al., 2020), for example, showed comparatively the most positive and long-term effects concerning time and effect.

In almost all doping prevention measures, instructors delivered the contents, such as teachers, coaches, or external trainers. Only the ethical decision-making training (Elbe and Brand, 2016) was based on completing work assignments in an online program. It can be assumed that this program did not offer the opportunity to ask questions or discuss program components, which means that there was no interactional part to the mediation. Only the ATLAS and ATHENA programs additionally use specifically trained peers as supporting instructors, who were supposed to ensure that the content was communicated and dealt with at eye level. The authors assessed this mediation as positive (e.g., Elliot et al., 2004). Otherwise, trained peers were also seen as a potential cause of variation in program implementation and possible limitations in effectiveness (Goldberg et al., 1996a; Yager et al., 2019).

Only two of the methods considered conveyed their content online: The ethical decision-making training (Elbe and Brand, 2016), which achieved a negative effect on doping attitudes after six sessions of 30 min each, meaning that the participants showed a more tolerant attitude in the posttest. Additionally, the iPlayClean program (Nicholls et al., 2020) was also tested in an online implementation. This program achieved a positive short-term and long-term effect on doping attitudes. Additionally, it led to a positive short-term effect on susceptibility (classified as resistance against offer in the results overview).

## Discussion

Doping is a significant problem in sport and can be countered by benefit-oriented education to protect young people from using performance-enhancing substances. Concerning the general effect, the short–if any–positive effects of doping prevention measures make it clear that there is much room for improvement. Regardless of the level of athletic performance, positive effects are essential in maintaining and promoting health. A long-term perspective links directly to building PL or HL. Literacy includes the elements required in the doping prevention context of access to reliable information, e.g., on forms of performance enhancement, together with an understanding of how to use it for one's benefit in the scope of informed decision-making and in accordance with one's context, such as elite or recreational sport (cf. Bröder et al., 2019). This also recognizes the maturity of young people. All prevention measures offer starting points to initiate personal development based on one's motivation and promote literacy. The multidimensional nature of the literacy constructs is conducive to this. Thus, learning about healthy nutrition and its performance-enhancing effect (e.g., Goldberg et al., 1996a) enables participants to improve their PL and HL. In the context of competitive sports, athletes also need permanent protection to avoid (accidentally) falling into doping traps. Focusing on elite sport, even annual prevention measures carried out by national anti-doping organizations do not seem to be enough regarding changes that can only be detected in the short term. Of course, the existing gray area and the difficulty of obtaining reliable data must be considered in this context (de Hon et al., 2015; Petróczi, 2016). Thus, consideration should be given to whether relying solely on self-report data is sufficient to make a robust assessment of interventions' impact.

Focusing on the target group, the increased consideration of young non-athletic target groups is in line with the perception of doping prevention as a societal issue (Petróczi et al., 2017) and perceiving the problem outside of competitive sport (Ahmadi and Svedsäter, 2016). If we compare the effects of doping prevention between athletic and non-athletic target groups, it seems more difficult to achieve positive effects in non-athletic target groups. Considering that adolescents also use doping substances outside the context of competitive sports, differentiation, and adaptation of the measures to the target group's needs are indicated. Thus, programs meet the demands for comprehensive doping prevention, also outside the realm of elite sport (e.g., Barkoukis et al., 2016). At this point, the target group of the prevention program is of particular relevance to the objective. While doping in competitive sports is prohibited behavior that can lead to sanctions and endanger the health of athletes, doping in recreational sports is a public health issue in which the areas of HL and PL are central. The success of a prevention program here is essential for the protection of health. In elite sports, it is a matter of broader objectives, including maintaining the integrity or credibility of individual athletes, federations, or the sport itself. Because of the frequent reference to a floor effect and low baseline rates (Ntoumanis et al., 2014; Lucidi et al., 2017; Yager et al., 2019), an adjustment could be made to focus on other target variables, such as the level of PL or HL. Considering the young age of initiation into using performance-enhancing substances (Nicholls et al., 2017), the age groups considered seem reasonable, if not too old, to prevent first use. Most studies are based on age groups of 15 years and older. Only the study by Laure et al. (2009) considers 10-year-olds. On the other hand, given the demand for early prevention, it seems logical that young target groups' effects are small. One can also take advantage of the fact that adolescent non-athletes are not subject to doping tests, including possible sanctions. This offers opportunities for prevention measures with positive connotations that focus on benefits (Englar-Carlson et al., 2016). While it is customary to take differentiation into account in school learning contexts (e.g., Australian Curriculum Assessment and Reporting Authority, n.d.), this possibility has been little used in the context of prevention measures. This is striking as elite athletes can also be considered as a heterogeneous group. Within a defined and modularized structure, only collaborative and active parts enable participants to realize and discuss their ideas.

Taking a closer look at the content, positive doping prevention approaches complement or even replaces measures that rely on repression, punishment, or scary stories. In line with Woolf (2020), the transmission of knowledge is not sufficient for the fight against doping. Ntoumanis et al. (2014) ideas for explaining the small effects of the two prevention programs ATHENA and ATLAS must also be considered. They see a possible explanation in the thematic breadth of the two programs (e.g., healthy nutrition, training) beyond the consideration of doping. In this respect, it seems beneficial to use the topic of doping and substance-induced performance enhancement as an overarching theme to which related topics can be specifically linked.

If we focus on the implementation of content, the cognitive domain of the ISE (World Anti-Doping Agency, 2021) seems dominant. This result is in line with the finding of Gatterer et al. (2019). The programs do not focus exclusively on the knowledge-focused approach and offer other elements such as life skills. This combination increases the practical relevance of the content for adolescents. In part, the composition of the program content seems arbitrary, also due to the lack of educational science findings (cf. Woolf, 2020) or the frequent omission of a sound theoretical derivation of the content and approach while “only” embedding it in the current state of research. Numerous researchers apply the TPB (Ajzen, 1991)–partly referring to the dominance of this theory in the context of doping and prevention. However, a mere reference to the frequency of use of a theory should not be used as the sole indicator of benefit in a given context and can potentially lead to distortions in perception, e.g., by frequently using TBP variables as indicators of the effectiveness of an intervention. A look at integrated models and the application of alternate ways to measure efficacy that go beyond self-report might be helpful (e.g., Lazuras, 2016; Petróczi, 2016).

Although a constructivist approach to learning is seen as having particular potential (Ertmer and Newby, 2013), cognitivist-dominated programs also achieve sound effects. For a more precise assessment, it is essential to look at long-term effects to assess whether only knowledge was acquired or whether deeper processing was also stimulated. For a clear statement on whether a constructivist understanding of learning is particularly successful or whether activating programs work better, more meaningful studies in terms of their research methodology would be needed.

Even short programs, such as the 60-min Clean Sport program (Hurst et al., 2020), have the potential to achieve substantial effects. This strategy supports anti-doping agencies' approach or recommendations to conduct workshops or booster sessions (Backhouse et al., 2012; World Anti-Doping Agency, 2021). It is therefore promising and time-efficient that short preventive measures lead to a benefit.

The integration of peers as tutors in the implementation of prevention interventions offers both opportunities and risks. On the one hand, there can be a discussion at eye level, and their use tends to be perceived positively; on the other hand, there seems to be an increased risk that they will not behave in a manner that is true to the manual. Manual fidelity is one of the essential criteria highlighted by Backhouse et al. (2012) to ensure that the program's intentions can work as planned. Integrating peers as tutors should be accompanied by didactical and methodological training on the program's background in addition to content training to enhance awareness and knowledge of central components of the doping prevention measure.

Online-based prevention can have a positive effect on athletes (Nicholls et al., 2020). Because of Elbe and Brand (2016) opposing effects, it seems essential that such implementations are accompanied by a regular evaluation so that the learning success can be documented and countermeasures can be taken if necessary. Given the topic's sensitive nature, especially for elite athletes, participants may have a greater anonymity feeling, leading them to deal more openly with the topics addressed. Besides, online-based implementations offer the possibility of increased individualization in the sense of greater freedom of choice regarding which topic is processed. This individualization is a step toward the recommending of tailored programs (Backhouse et al., 2012). Avatars could be implemented to increase collaborative parts in the sense of a constructivist understanding of learning and joint topic editing, “who” respond to the participants' answers. Especially in the area of the affect-based approach and values-based education, the confrontation with others and social embedding could be an improvement and beneficial for participants.

The studies considered differ considerably concerning methodological research quality (see Table 4). This weakness is also shown by the mean assessment of study quality, including a comparatively large standard deviation. In most studies considered, no power analysis was performed in advance to determine the optimal sample size. Furthermore, there is often a lack of report whether the data quality justifies parametric procedures (e.g., normality, homogeneity of variance). The specification of effect sizes and confidence intervals would also help interpret the results in terms of their significance. Unfortunately, this information can only be found in a few studies (Horcajo et al., 2019).

The review has some limitations. First, only doping prevention measures whose results were published in peer-reviewed journals are included. Gatterer et al. (2019) describe the prevention content of 53 national anti-doping organization's programs in their analysis. It is striking that most of these efforts were not included in the review. It can be assumed that evaluations of the programs are conducted internally. However, these data are not included due to the lack of publication in scientific journals. The blind spots that result here could be addressed through coordinated and collaborative planning, implementation, and evaluation of doping prevention efforts in multidisciplinary teams of scientists and practitioners (see Woolf, 2020). The ISE (World Anti-Doping Agency, 2021) represents a promising step in this direction. Second, the ATLAS program is overrepresented in analyses due to the comparatively large number of publications referencing this approach (e.g., Goldberg et al., 1996b, 2000; Yager et al., 2019). However, this also has the benefit of revealing that the program does not produce consistent results (see Table 4). Third, new issues such as the protection of clean athletes have hardly played a role in the prevention efforts. In competitive sports, there is already a fine line between offense-based prevention and general suspicion. Recent developments in this direction (e.g., Englar-Carlson et al., 2016; Petróczi et al., 2021) could make a constructive contribution to athletes in competitive sport contexts and society as a whole and should therefore be given more significant consideration.

## Practical Implications for Prevention

The beginnings in doping prevention have been made for more than 30 years, yet there is much room for improvement. Existing programs and prevention ideas are designed to protect and promote young people outside of competitive sports in terms of their health. In the elite sports context, athletes should learn early on what behavior is prohibited. The following aspects complement previous recommendations for the design of effective doping prevention measures (e.g., Backhouse et al., 2012):

(a) Doping prevention measures should be scientifically monitored and evaluated in longitudinal or experimental designs. This approach implies the need for long-term research funding opportunities that also include consideration of follow-ups (e.g., Boardley et al., 2021).
(b) More international and transdisciplinary collaborative doping prevention networks composed of researchers and individuals from elite sport (e.g., athletes) should be established (Boardley et al., 2021).
(c) In terms of tailored doping prevention measures, developers of anti-doping interventions should consider a modular system that offers participants opportunities for differentiation within an overarching theme. This differentiation should increase interest and enable more efficient learning.
(d)In addition to self-reporting, alternative methods like implicit or indirect procedures should be used to consider the effects of doping prevention measures (Petróczi, 2016).
(e)Online-based prevention interventions offer benefits of increased individualization but should be evaluated in terms of learning success.
(f) Prevention measures should be integrated into school curricula at an early stage and with a positive connotation (possibly also through interdisciplinary projects in order to consider the interests of young people better) so that a constructive atmosphere can be created.
(G) The perspective on clean athletes and their empowerment should be expanded (Englar-Carlson et al., 2016; Boardley et al., 2021).

In order to assess the success of doping prevention measures, evidence-based monitoring and evaluation for the long term are essential. This allows the identification of functioning prevention components, which should also have a beneficial effect on the compliance of students and athletes. To sum up, we see a necessity to implement doping prevention measures for young target groups outside the elite sport context. Focusing on the empirical results of current implementations, we see favorable outcomes, room for further improvement in theory and practice, and a need for evaluation and publication of results.

## Data Availability Statement

The datasets presented in this study can be found in online repositories. The names of the repository/repositories and accession number(s) can be found in the article/supplementary material.

## Author Contributions

The author confirms being the sole contributor of this work and has approved it for publication.

## Conflict of Interest

The author declares that the research was conducted in the absence of any commercial or financial relationships that could be construed as a potential conflict of interest.

## Publisher's Note

All claims expressed in this article are solely those of the authors and do not necessarily represent those of their affiliated organizations, or those of the publisher, the editors and the reviewers. Any product that may be evaluated in this article, or claim that may be made by its manufacturer, is not guaranteed or endorsed by the publisher.
